# R-HPDC Process with Forced Convection Mixing Device for Automotive Part of A380 Aluminum Alloy

**DOI:** 10.3390/ma7043084

**Published:** 2014-04-15

**Authors:** Bing Zhou, Yonglin Kang, Mingfan Qi, Huanhuan Zhang, Guoming Zhu

**Affiliations:** School of Materials Science and Engineering, University of Science and Technology Beijing, Beijing 100083, China; E-Mails: zb521a@sina.com (B.Z.); qimfan@163.com (M.Q.); 1989zhanghuanhuan@163.com (H.Z.); zhuguoming@ustb.edu.cn (G.Z.)

**Keywords:** forced convection mixing device, semi-solid, die casting, fluid convection, A380 aluminum alloy, porosity, casting defects

## Abstract

The continuing quest for cost-effective and complex shaped aluminum castings with fewer defects for applications in the automotive industries has aroused the interest in rheological high pressure die casting (R-HPDC). A new machine, forced convection mixing (FCM) device, based on the mechanical stirring and convection mixing theory for the preparation of semisolid slurry in convenience and functionality was proposed to produce the automotive shock absorber part by R-HPDC process. The effect of barrel temperature and rotational speed of the device on the grain size and morphology of semi-solid slurry were extensively studied. In addition, flow behavior and temperature field of the melt in the FCM process was investigated combining computational fluid dynamics simulation. The results indicate that the microstructure and pore defects at different locations of R-HPDC casting have been greatly improved. The vigorous fluid convection in FCM process has changed the temperature field and composition distribution of conventional solidification. Appropriately increasing the rotational speed can lead to a uniform temperature filed sooner. The lower barrel temperature leads to a larger uniform degree of supercooling of the melt that benefits the promotion of nucleation rate. Both of them contribute to the decrease of the grain size and the roundness of grain morphology.

## 1. Introduction

Eutectic and near-eutectic Al-Si alloys are used extensively in the casting industry mainly due to their high strength to weight ratio, excellent abrasion and corrosion resistance, low coefficient of thermal expansion [1–3]. These properties contribute to the application of Al-Si alloys in the automotive industry, especially for cylinder blocks, cylinder heads, pistons and valve lifters [4].

The Al-Si based alloys are transversally used in different foundry processes. Among all the technologies, high pressure die casting (HPDC) is the most widely used one in the process of producing automotive components. It is a cost-effective process that produces parts with complex geometry and accurate dimensions as well as good surface finish [5,6]. However, parts made by using HPDC generally suffer from porosity resulting from gas entrapment and solidification shrinkage which, along with defects caused by inclusions contained in the melt [7–9], have traditionally limited the scope for weight reduction in these castings. Therefore, there have been considerable efforts to minimize these problems by introducing more advanced shaping routes such as rheoforming and thixoforming [10–12].

Semi-solid metal forming has been applied in HPDC process for more than 30 years to solve these problems and it has already brought other benefits such as die life extension [13]. In the thixo-die casting process, the material is treated in such a way that the microstructure will be spheroidal in the semi-solid state; a slug of alloy is heated up to reach the required semi-solid state, transferred to a shot system and then injected into the die. Improved quality with high mechanical properties has been reported [14,15]. However, it is quite difficult and costly to form semi-solid metal in the slug form and the process requires major modifications to the machine and process [16]. Therefore, recent studies have shifted the focus to injection of semi-solid slurries at low solid fractions since semi-solid slurries can be directly poured into the current shot system. The aim of R-HPDC process is to reduce the cost of the system and the needs to modify the machine, die and processes. The process also creates several cost benefits such as cycle time reduction, lubricant usage reduction and melting energy reduction.

The main requirement for the success of R-HPDC process is the production of a non-dendritic, spheroidal microstructure, from which suitable viscosity and adequate flow behavior are expected. So far, many semi-solid slurry preparation technologies have been proposed, such as mechanical stirring, electromagnetic stirring [17], continuous rheoconversion process [18], low superheat pouring with a shear field process [19], gas induced semi-solid process [20], serpentine channel pouring process [21]. Many semi-solid processes based on mechanical agitation principle have been proposed. The first rheomoulding prototype is a vertical injection and vertical clamping single screw machine patented by Cornell University in 1993 [22]. Fan and co-workers [23] have developed a twin screw rheomoulding process in Brunel University in 1999. Kang [24] has developed the taper barrel rheomoulding process with stirring inner tube for semisolid slurry preparation in USTB in 2008. Kang [25] has developed a process proposal for continuous fabrication of rheological material by rotational barrel with stirring screw in Pusan National University in 2009. These processes are the most widely used in the lab. However some disadvantages such as easy sticking, blocking up matter, troublesome cleanup and complex disassembly and installation exist widespread in these kind of device. Furthermore, the preparation of semi-solid slurry is unstable due to the crawl space and the direct contact with the metal walls.

One of the promising techniques for semi-solid slurry preparation focusing on stability and convenience is the forced convection mixing (FCM) device developed by Kang *et.al*. [26]. The FCM device has exquisite compact structure. Multiple high purity graphite parts have been used to avoid sticking matter on the wall. The device is easy to maintain because the disassembly, clean-up and assembly are simple, only costing two people half an hour. In addition, the easily-worn parts can be replaced conveniently. Some different kinds of satisfactory semi-solid slurry of light alloys have been prepared, such as 7075 aluminum alloy, A356 aluminum, A380 aluminum and AZ91D magnesium alloy.

In the present research, a typical A380 foundry alloy part of automotive shock absorber is prepared by R-HPDC process combined with FCM device. The study investigates the microstructure and porosity of rheological castings in different locations. The effects of process parameters on semisolid microstructure are also investigated. Understanding the flow behavior and temperature field of the melt is the key to figure out the crystallization of semisolid slurry in FCM process. Computational fluid dynamic (CFD) simulation helps to reproduce the fluid convection and temperature field variation of the melt in FCM device. The effects of process parameters on nucleation and grain growth of semisolid slurry combining CFD results were discussed.

## 2. Results

### 2.1. Microstructure and Pore Defects of A380 R-HPDC Part

**Figure 1.**
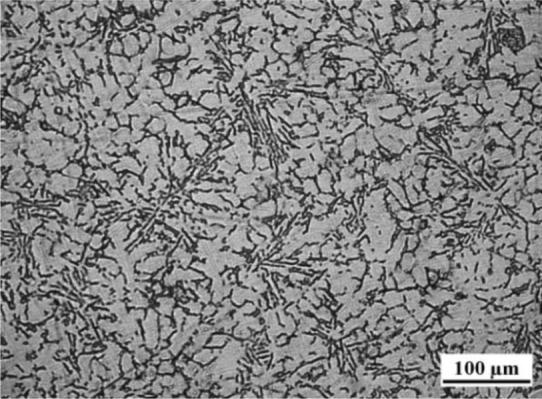
As-cast microstructure of A380 Al alloy during conventional solidification.

Figure 1 shows the as-cast microstructure of A380 Al alloy prepared by conventional HPDC process with the pouring temperature at 640 °C. The white phase is primary α-Al and the developed dendrites are observed.

**Figure 2.**
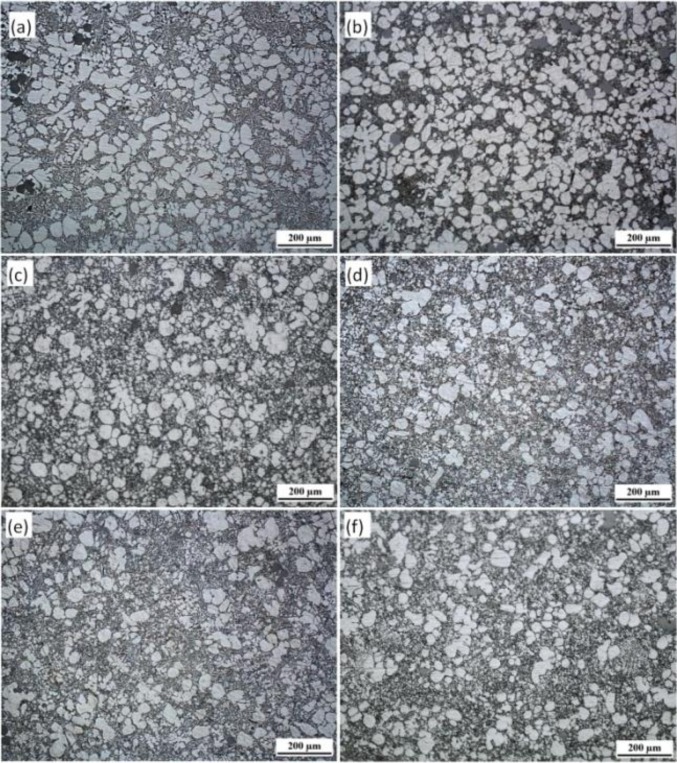
Microstructure distribution of R-HPDC automobile shock absorber part at each point (**a**–**f**).

Figure 2 shows the microstructure from the different parts of the R-HPDC automobile shock absorber part. The parameters of FCM device is the pouring temperature 610 °C, the barrel temperature 560 °C, rotational speed 300 r/min and mixing time 30 s. It can be seen that the microstructure of the R-HPDC component is mainly composed of rosiness and nearly spherical primary α-Al particles and few dendrites can be found. The primary mean particles size is about 60 μm. The microstructure at the position A mainly consists of rosiness and random-shaped primary particles. The structure morphology is relatively coarse. The reason is that the heat dissipation of the melt mainly relies on one-way heat exchange with the shot sleeve and the large size of biscuit needs a long solidification time. So some approximate spherical grains at the biscuit formed in the FCM device grow in conventional solidification. The microstructure at the point B in the runner is improved. The quenching effect of die cavity on the generation of new particles is obvious, with significant increases in the number of the particles. The melt in the runner need a shorter solidification time due to the thinner thickness and the morphology of particles is relatively rounder. All the microstructure at the point C, D, E and F in the actual part has small and nearly spherical particles distributing evenly through the matrix. The microstructure in different positions is slightly different. Small dendrites can be found at the point C. Primary α-Al particles at point F are relatively smaller and rounder than the particles at point C.

The proportion of solid and liquid phase in different positions is affected by die casting filling process. The solid content of semisolid part at the point A, B is more than the point E, F. The reason is that the semisolid slurry has larger apparent viscosity than the ordinary melt. And the primary particles and the liquids of the slurry have different flow speed during the filling process [27]. The liquids moves faster and there are more liquid phase in a relative distant place of parts.

**Figure 3.**
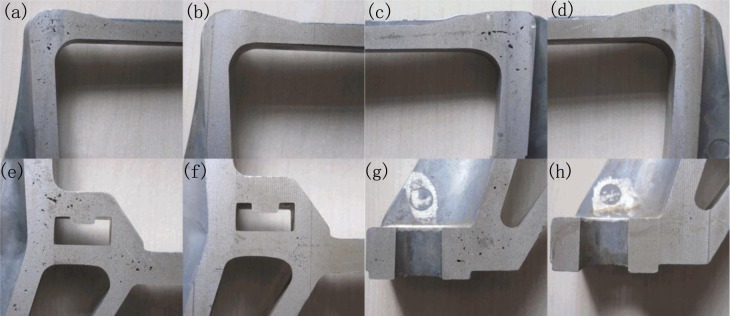
Pore defects of automobile shock absorber part in different locations by (**a**,**c**,**e**,**g**) traditional HPDC process and (**b**,**d**,**f**,**h**) R-HPDC process.

Automobile shock absorber part in use is wearing parts and the casting defects has serious impact on the mechnical properties and sevice life. The R-HPDC process can signficiently improve casting defects, especially pore defects. Figure 3 shows the pore defects on the cross sections of componets in different locations under HPDC and R-HPDC process. It is obvious that a lot of pore defects exists on the cross sections of traditonal die castings and very few pore defects exists on the cross sections of rheological die castings. The serious difference of pore defects between castings is not only in the quantity but also in the size.

**Figure 4.**
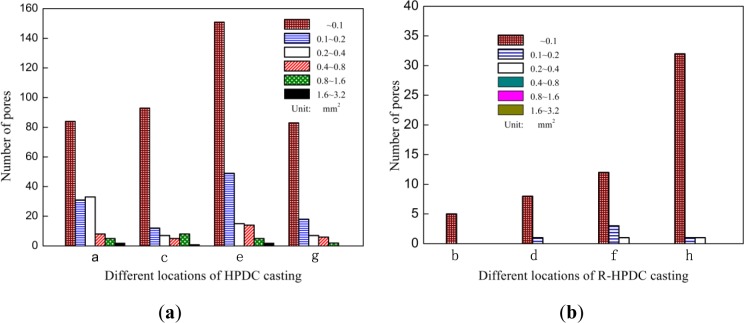
Number of pores in different locations of A380 parts by (**a**) HPDC and (**b**) R-HPDC process.

The image processing software was used to measure the area of pores in different locations (a~h) in Figure 3. Figure 4 shows the comparison of the exact number of pores in different locations of A380 automobile shock absorber part by HPDC and R-HPDC process. The conventional HPDC casting has a large number of pore with dimensions from pinholes to 3.2 mm^2^. The number of the pores in HPDC casting decreases with the size. The R-HPDC process has obviously decreased the number of pores in the castings. On one hand, the number of pinholes are largely reduced and the nuber of large pores are basically eliminated especially for the cross-sectional area above 0.4 mm^2^. On the other hand, the total cross-sectional area of pore defects from four parts of HPDC casings, shown in Figure 3, is about 101.32 mm^2^ and the total cross-sectional area of pore defects from four parts of R-HPDC castings is about 3.58 mm^2^. Compare to the HPDC process, The R-HPDC process with the FCM device has decresed the total cross-sectional area of pore defects by about 27 times in one of the cross section.

### 2.2. Effects of Barrel Temperature on Semisolid Microstructure

**Figure 5.**
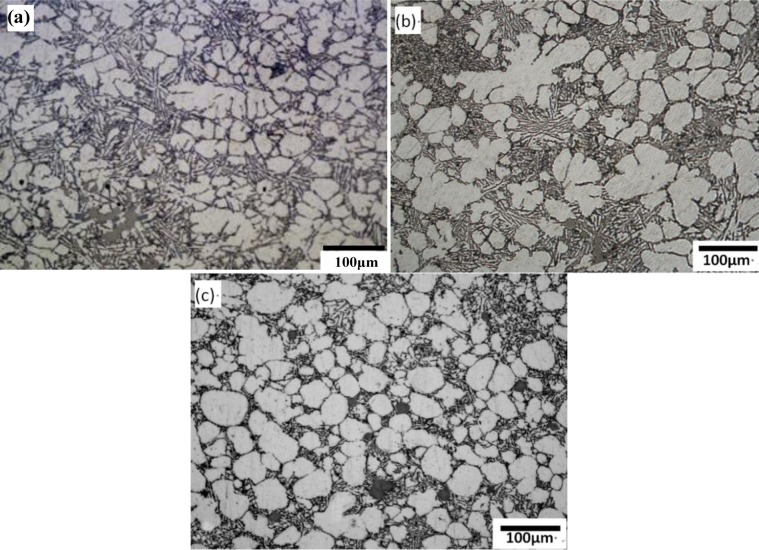
Optical micrographs of semisolid A380 alloy under different barrel temperature (**a**) 580 °C; (**b**) 560 °C; (**c**) 540 °C.

Figure 5 shows the optical microstructures in the biscuit of the rheo-diecasting A380 Al alloy under different barrel temperatures. The other parameters of the FCM process were the pouring temperature at 610 °C, the rotational speed at 300 r/min and the mixing time in 30 s. It reveals that all the microstructures of semisolid Al alloy with the FCM process consists of spherical or rose-like primary α-Al particles and little dendrites can be found, which is obviously different from the microstructure of traditional liquid alloy. When the barrel temperature is 580 °C, the microstructure mainly includes rosiness particles and small dendrites. When the barrel temperature is 560 °C the microstructure mainly includes rosiness particles and the size of primary particles is decreased. When the barrel temperature decreases to 540 °C, the amount of primary particles have greatly increased and the morphology is obviously refined. The average particles size is about 57 μm and the shape factor is close to 0.9. Meanwhile, with the decrease of the barrel temperature, the number of the primary particles is increasing, caused high solid fraction.

**Figure 6.**
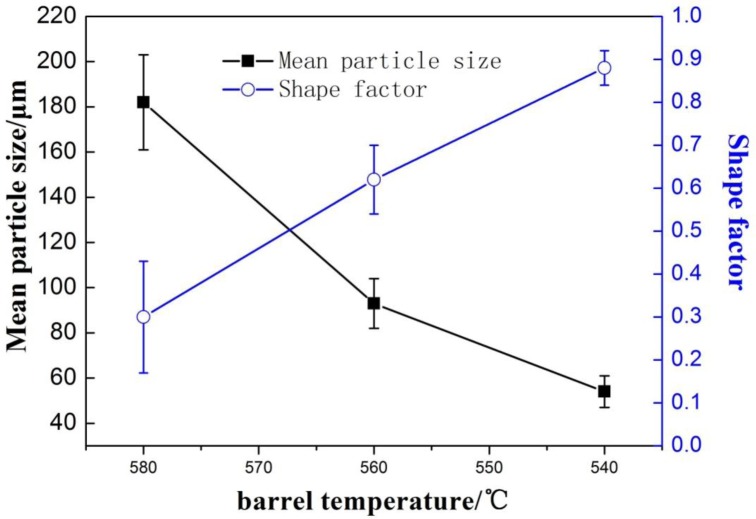
Variations of primary particle mean size and shape factor with barrel temperature.

When the pouring temperature of the melt is certain, the lower barrel temperature of the device would lead to the melt larger intensity of cooling. Figure 6 shows the mean particle size and the shape factor of primary particles at different barrel temperatures. When the barrel temperature is 580 °C, the cooling effect is not obvious enough and the number of generated grains is small. With the decrease of barrel temperature, the intensity of cooling is enhanced and the temperature of the molten alloy can be dropped down in the semisolid region through convective heat transfer. When the barrel temperature is 540 °C, the melt can reach the largest degree of supercooling in shorter time. At last the microstructure has the largest amount of crystal grains and the morphology is the roundest one compared with others. Meanwhile, From Figures 5 and 6, it can be summed up that lower barrel temperature is beneficial to decrease the mean particle size and improve the crystal grains’ morphology.

### 2.3. Effects of Rotational Speed on Semisolid Microstructure

**Figure 7.**
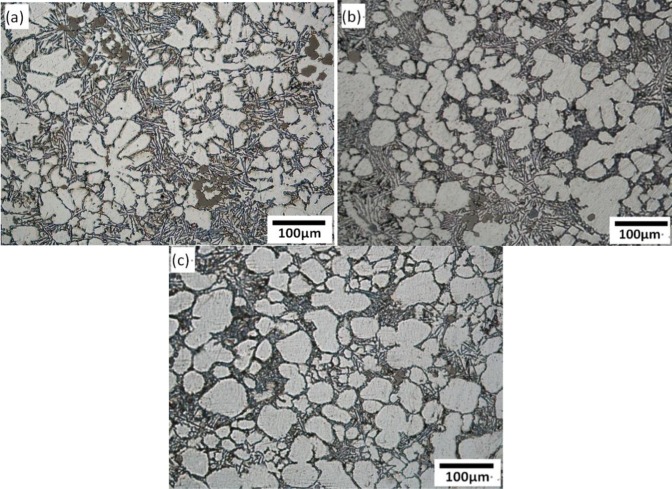
Optical micrographs of semisolid A380 alloy under different rotational speed: (**a**) 100 r/min; (**b**) 300 r/min; (**c**) 500 r/min.

Figure 7 shows the optical micrographs in the biscuit of semi-solid part under different rotational speeds. The other parameters of the FCM process were the pouring temperature at 610 °C, the barrel temperature at 560 °C and the mixing time of 30 s. When the rotational speed is 100 r/min, the microstructure has many larger rosiness particles and smaller dendrites. The number of the particles is small. When the rotational speed is 300 r/min, the amount of primary particles is increased and the morphology of most particles has been improved and small dendrites are roughly invisible. With the further increase of rotational speed, desirable primary particles are distributed in the matrix uniformly. The average particles size is about 70 μm and the shape factor is above 0.75.

**Figure 8.**
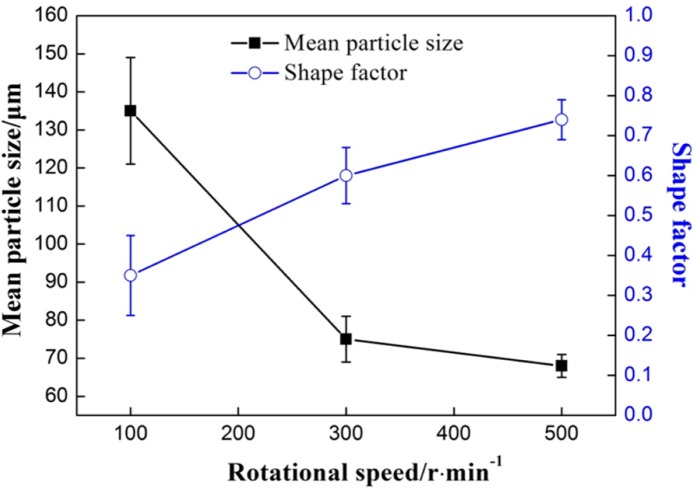
Variations of primary particle mean size and shape factor with rotational speed.

Figure 8 shows the variations of mean primary particle size and shape factor of primary particles under different rotational speeds. From the Figures 7 and 8, it is obvious that with the increase of rotational speed, the mean size of the α-Al particles diminishes and the shape factor increases. The primary particles become fine and homogeneous.

The rotational speed of the device is directly related to the intensity of mixed convection. When the rotational speed is low, the convection intensity of the melt is low. On the one hand, according to traditional crystal dissociating theory [28], some grains adhered to the wall and helical blade to nucleate are difficult to free to the melt because of the low shearing rate. On the other hand, it needs more time to reach the uniform temperature distribution in the melt and there is a big temperature difference inside the melt for a certain time, which is conductive to the growth of globular grains but dendrites. With the increase of rotational speed, the intensity of mixed convection is enhanced and the uniform temperature distribution can be achieved in a short time. The small temperature gradient is beneficial to improve the morphology of primary particle. Furthermore, some grains adhered to the wall to nucleate are easier to free to the melt. It can increase the amount of grains and enhance the mutual inhibition between the grains.

### 2.4. Computational Fluid Dynamic Results

The research of flow characteristic in FCM device will be helpful to understand the way of heat transfer and the change of temperature, and thus provides theoretical basis for adjusting process parameters and controlling solidification process.

**Figure 9.**
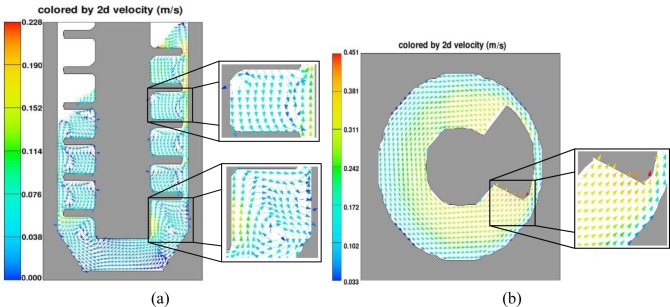
Flow characteristics simulation of the melt in FCM device (**a**) axial velocity vector; (**b**) circular velocity vector.

The melt in FCM device has complex stirring-mixed flow characteristics. As shown in Figure 9, the velocity vector of the fluid in three dimensions can be decomposed into the axial velocity vector and the circular velocity vector in 2D pattern for flow analysis in FCM device. From Figure 9a, we can clearly see that the axial velocity vector has two parts: internal axial velocity vector from high to low under the compression of helical blade and gap velocity vector from low to high due to the reaction of axial flow. Meanwhile the melt has the circular velocity vector in the same direction with the rotation direction as shown in Figure 9b.

**Figure 10.**
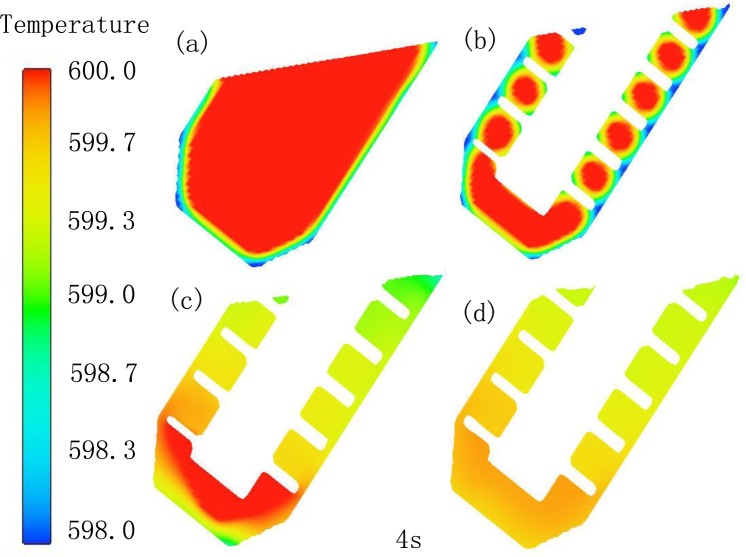
Temperature field simulation of the melt in FCM device at time 4 s: (**a**) without agitating shaft; (**b**) 0 r/min; (**c**) 100 r/min; (**d**) 300 r/min.

The melt has sufficient convection in three dimensions, the temperature field of the melt in the device will be affected enormously. Figure 10 shows the temperature field simulation of the melt in different conditions. The initial simulation parameters are the melt temperature 610 °C, the graphite barrel temperature 560 °C and the temperature of agitating shaft 560 °C. In Figure 10a, there is huge temperature difference in the whole melt, with higher temperature inside and lower outside. In Figure 10b, the introduction of the agitating shaft results in the temperature decreased in the melt. However, the big temperature difference still exists in the melt between the ribbons because of zero rotational speed. With the rotational speed 100 r/min, the distribution of the temperature field has greatly changed due to fluid convection. There is no significant temperature difference of the melt at the top of the device. The big temperature difference still exists at the bottom due to weak convection caused by the position. The uniform temperature distribution can be improved through increasing rotational speed, especially at the bottom. In addition, the temperature of the whole melt is about 599 °C at time 4 s, which is nearly remaining constant to the end. However, the outlet temperature of the semisolid slurry under the same parameters is about 500 °C in the practical experiments. The temperature difference between the simulation results and practical experiments is about 9 °C. The reason is that the simulation does not consider the heat loss of the melt during the pouring process.

**Figure 11.**
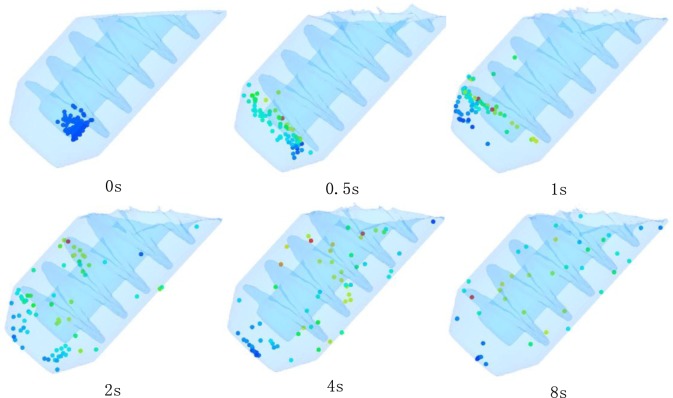
Distribution of the particles in the melt at different time.

The change of the melt caused by the vigorous fluid convection is not only the temperature field, but also the composition distribution. During the simulation of stirring process, random tracer particle method is adopted [29], which considers the effects of fluid convection in the device on the distribution of the composition. The simulation results are shown in Figure 11. It can be seen that with the action of stirring shaft, the original massed random particles gradually spread apart over time and finally evenly distribute in the melt.

## 3. Discussion

The purpose of the discussion part is to demonstrate the solidification of R-HPDC process and the influence of fluid convection on the formation of semi-solid microstructure in FCM device.

During traditional HPDC process most of the nuclei relying on the wall of shot sleeve and mold are generated due to the rapid cooling. When the nuclei enter into the flowing melt, some are melted by superheated temperature and others are survived. Only a small amount of grain exists in the final components and the microstructure is mainly dendrites. The turbulent fluid flow in filling process could easily lead to oxide scale and large volume of air entrapment [30]. The high pouring temperature could increase the internal solidification time of die castings and shrinkage defects often appear in the center especially for a slightly thicker casting.

**Figure 12.**
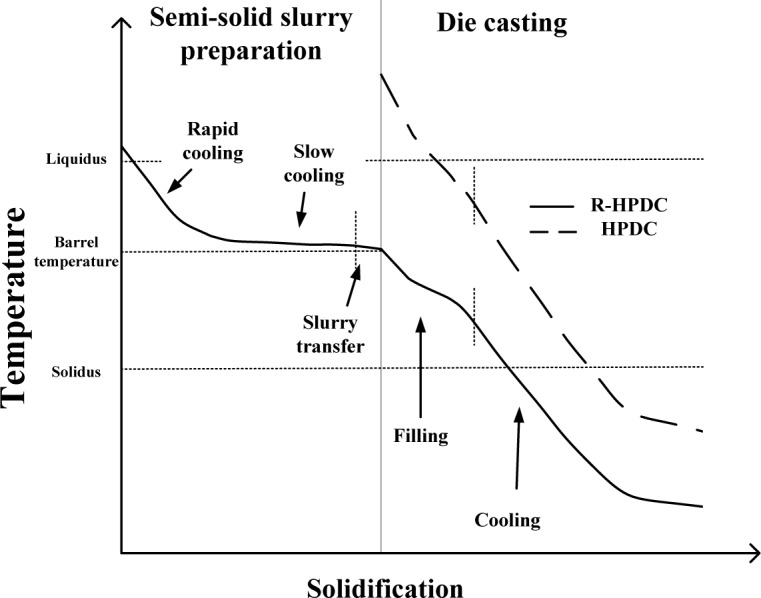
Solidification process of the melt in traditional HPDC process and R-HPDC process.

Figure 12 shows the different solidification process of the melt in traditional HPDC process and R-HPDC process. The R-HPDC process consists of two processes: semi-solid slurry preparation and die casting process. Take the FCM device for example. Under the action of gravity and stirring rod equipped with helical blade the molten alloy has complex flow characteristics. The melt has to go through the rapid cooling, slow cooling and slurry transfer stage. Because of the suitable barrel temperature within the range of solidus and liquidus, it can remain in semisolid temperature interval for a long time, which is beneficial to generate a large number of crystal nucei. Along with the action of flow convection, the temperature field of the melt in the device becomes uniform and the environment for dendrite growth is destroyed. Finally most nuclei grow to approximate spherical grain. Then transfer the prepared semi-solid slurry to the shot sleeve and carry out the die casting process. There are significant differences between the semi-solid slurry and the normal melt during die casting process, which results in different performance of porosity on the cross section. Firstly, there is a tendency of mutual restrain between amount grains in subsequent solidification of semisolid slurry. The refined and homogeneous crystal grains instead of dendrites in final rheological die castings will improve the performance. Secondly, the semi-solid slurry has unique performance of high viscosity and good fluidity. It can reduce the turbulence and splash induced as the fluid flows and tend to smooth flow in filling process. The entrapped gas content decreasing in shot sleeve and die-casting mold will effectively reduce porosity of castings. Thirdly, the semi-solid slurry with lower forming temperature leads to small solidification shrinkage, which can significantly reduce shrinkage defects especially in the thick wall place.

Also, the parameters of conventional HPDC such as slow shot and fast shot as well as up set pressure have improtant influence on the porosity. Verran’s results demonstrated that high speed of slow and fast shots and low-up set pressure result the worst density and maximum porosity [31]. The resuts are in agreement with theoretical and experimental predictions about the influence of speed of slow and fast shots on formation of porosity [32,33]. The use of high speed for the slow shot, as well as for the fast shot, increases turbulence in liquid metal, thus causing air entrapment by the filling front and formation of porosity in diecasing parts. Regarding the up set pressure, the hypothesis of high pressure, in order to obtain the optimum casting density for the parts [34].

The R-HPDC castings without shrinkage and porosity have another advantage: heat-treatable. Heat treatments to die casting aluminum alloys were seldom studied since high temperature triggers severe gas expansion and irreversible dimensional variations [35,36], both of which are unacceptable for die casting products. One of the main reasons is porosity contained in die casting products. As mentioned previously, rheological die casting process greatly improves the porosity defects as shown in Figure 5. The dimensional variations and surface bubbling phenomena do not appear after the appropriate T6 heat treatment, for rheological die castings, and better comprehensive mechanical properties can be achieved [37–40]. The R-HPDC process has a bright prospect and the applications in the automotive industry might be further expanded.

CFD simulation offers a powerful and cost effective way to study the effectiveness of different die designs and filling processes. There are a number of available methods and software packages for casting simulation and analysis [41,42]. These packages are grid-based and generally employ the volume-of-fluid (VOF) method to track the free surfaces, such as Flow-3D and Procast. Although they adopt different grid methods of finite volume method (FVM) and finite element method (FEM) to discrete data, the filling processes of semisolid slurry can be realized both with non-Newton viscosity modules. The FEM and FVM method can both realize the mixing process. In this paper, the simulation of semisolid slurry preparation was realized by FVM because it is uncomplicated and easy to implement.

**Figure 13.**
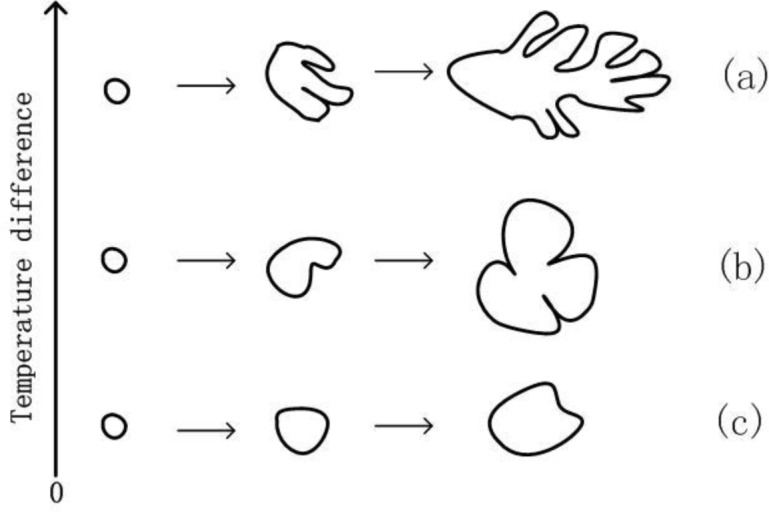
Schematic illustration of grain growth with temperature difference around the grain.

The quality of final rheological die castings is affected by the semi-solid slurry quality, especially the number and morphology of grains. Once generated, the stable nuclei enter the growth phase and the final morphology of the grains is primary influenced by temperature field. From Figures 9 and 10, the temperature field of the melt in FCM device is enormously affected by fluid convection. The uniform temperature distribution can be achieved with suitable process parameters, which is different with the huge temperature difference from traditional solidification. Figure 13 shows the schematic illustration of grain growth with the temperature difference around the grain. During the traditional solidification, there is only unidirectional heat transfer to the mold within the melt. Larger temperature gradient exists in the melt and dendrite arms are more inclined to develop in the direction of the lower temperature or the larger undercooling. The nuclei are finally transformed into fully developed dendrites as shown in Figure 13a. In FCM device the temperature field around the grain has changed by fluid convection that makes unidirectional temperature gradient completely disappear. The efficiency of heat exchange is obvious increased through forced convection heat transfer. The relative uniform temperature distribution can be available in a given time and small temperature difference also exists around the grain. The nuclei are generally grown to rosiness grains as shown in Figure 13b. Rotational speed is directly related to the strength of fluid convection. Increasing the rotational speed can obviously improve temperature distribution and reduce temperature difference around the grain within the same time. Furthermore, the time of grain getting into a state of uneven temperature distribution is significantly decreased and the directional growth of grains is weakened. In this case it is conductive to the generation of approximate spherical grain, as shown in Figure 13c.

Grain size is an important factor to affect the metal performances. General speaking, materials with fine grain structure have high comprehensive mechanical properties. Grain size is mainly affected by two factors: the nucleation rate and the duration time of crystallization. If the generation of the nucleation rate is faster, and the duration time of crystallization is longer, it generates more cell number, so the grain is fine. According to classical solidification theory [43], for homogeneous nucleation and heterogeneous nucleation, the critical nucleation radius is inversely proportional to the degree of supercooling and the critical nucleation energy varies inversely as the square of the degree of supercooling, so lower degree of supercooling helps increase the nucleation rate. With the fluid convection changing the temperature field, the whole melt is in a super-cooled state during the FCM process. The generation of new nuclei can appear anywhere in the melt and this will greatly increase the quantity of grains. Furthermore, the melt can achieve a larger degree of supercooling for further promotion of the nucleation rate through the appropriate decrease of barrel temperature of the process. Similarly, the decrease of pouring temperature of the process, lower degree of superheat actually, can make the melt of the device quick in a supercooling temperature range and nucleation more and faster in a limited time for semi-solid slurry preparation. On the other hand, the mixing time of the device affects the duration time of crystallization. More nuclei can be obtained by extending the mixing time. However the semi-solid slurry is also facing the risks, such as excessive grain growth and involved gas. So the mixing time of the process should be reduced appropriately while getting the satisfactory semi-solid slurry.

The vigorous fluid convection in FCM process has also changed the composition distribution of conventional solidification. In traditional solidification process the melt near the colder wall of the mold first solidify in high purity, then the center with high level of alloying elements and impurity elements solidify. The uneven chemical composition in a large area often appears and it is difficult to eliminate using later process [44,45]. For the FCM process, with fluid convection the newly generated and existing nuclei are constantly moving and gradually uniformed distribution in semi-solid slurry, as shown in Figure 11. Meanwhile the alloying elements in the melt can be completely mixed and become uniform. The macro-segregation can be improved, whether the semisolid slurry is used for ingot preparation or rheological forming.

## 4. Experimental Procedures

**Figure 14.**
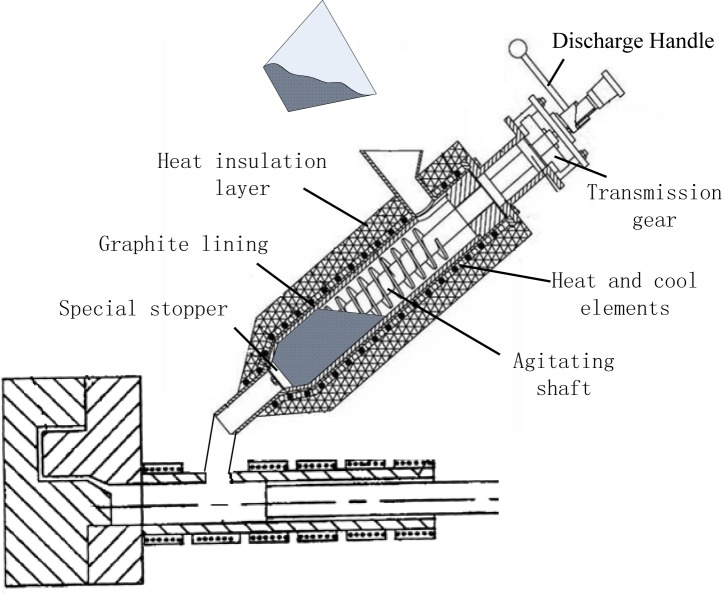
Schematic illustration of FCM-RHPDC process.

The self-developed FCM device mainly consists of the shearing system, the transmission system, the heating and cooling elements, the PID temperature controller, the discharge system and so on. The shearing system mainly includes the agitating shaft equipped with helical blade. The heating and cooling elements are wrapped around the wall of stainless steel barrel to ensure a constant temperature with the PID temperature controller. The working of discharge system is realized through discharge handle and graphite blockage connected by the core pin. The mixing chamber is equipped with graphite lining for improving sticking matter. The schematic illustration of the forced convection mixing rheological die casting (FCM-RHPDC) process is shown in Figure 14.

**Figure 15.**
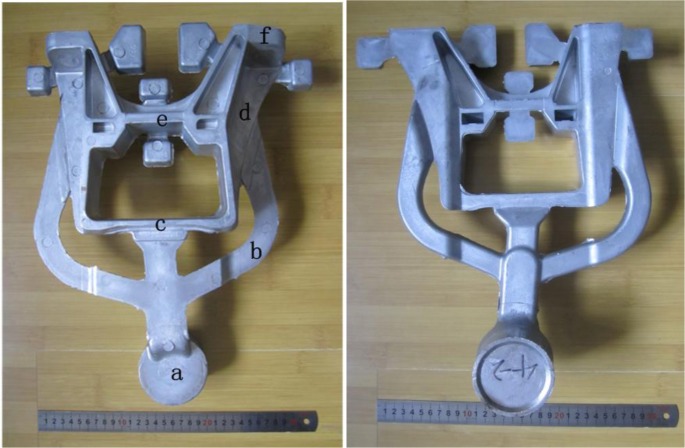
R-HPDC automobile shock absorber part.

The experiment material was commercial A380 aluminum alloy with the chemical composition shown in Table 1. The liquidus temperature of this alloy is 595 °C and the solidus temperature is 521 °C. The alloy ingot was made dried and then put into a melting furnace kept at 660 °C. After refinement and degassing treatment at 700 °C, the molten alloy was set aside about 30 min for cooling down to the desired pouring temperature. The inner barrel temperature of the device was controlled between 540 and 580 °C and the rotational speed was about 100–500 r/min. About 2.8 kg of the melt was poured into the chamber of the device. It was fully mixed through the stirring blade. After being stirred for 30 s, prepared semi-solid slurry was discharged by raising the graphite blockage and then transferred and shaped by a cold-chamber die-casting machine (HPDC machine) to produce the automobile shock absorber part. The mold temperature is above 180 °C. For comparison, conventional die casting samples were also made at a pouring temperature of 640 °C. The actual semi-solid automobile shock absorber part and the specific positions were shown in Figure 15. The microstructure from different positions of the R-HPDC part was analyzed. In addition, the effects of the barrel temperature and the rotational speed on the microstructure of semi-solid slurry were analyzed. Specimens for metallographic examination were cut from the biscuit, then polished and etched by 0.5% hydrofluoric acid solution. The parts prepared by R-HPDC process and traditional die casting process were cut crosswise into two pieces to compare the cast pore defects. The equivalent diameter (*d*) and shape factor (*f*) of primary phase are calculated using the following formulas:

$$d=\sqrt{\frac{4A}{\pi }}$$ (1)

$$f=\frac{4\pi Α}{P^{2}}$$ (2)

where *A* and *P* are the area and perimeter of particle, respectively. Based on the measurements, the area and perimeter of primary particles appeared on the metallographic graphs of several samples were measured by image processing software. The exact value of every primary particle can be measured by the ruler on the graphs.

## 5. CFD Simulation Procedures

The CFD simulation procedures of the fluid stirring in FCM device are as follows: First, the 3D solid model with actual size is created by Pro/E software. Second, the standard joint form IGES file is output. Third, the above model is transmitted to Meshing–Geometry Block in Flow-3D software. Then, the attributes of material, heat exchange coefficient on boundary, rotational speed, initial temperature and other Pre-Processing parameters are set. After the calculation, simulation results are directly observed through the Post-Processing block.

**Figure 16.**
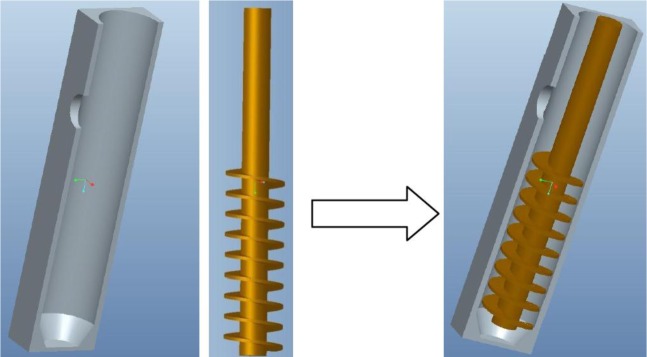
Simplified 3D model of FCM device.

**Figure 17.**
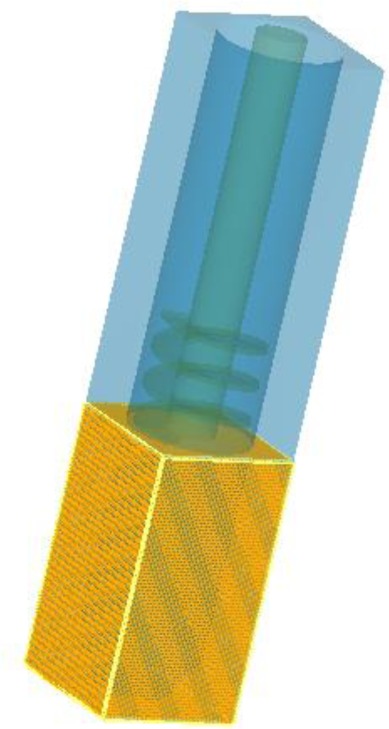
Mesh generation of simplified FCM model.

The effect of rotational speed on the flow characteristics and temperature field of the melt in FCM device is important to understanding the nucleation and grain growth mechanism. Numerical simulation was carried out with simplified model of real size as shown in Figure 16. The flow behavior of the melt in FCM process could be divided into pouring and stirring process. The simulation only considered the stirring process to reduce the complex influence of pouring process, which took about one second. The finite difference method was used to generate the hexahedral mesh of simplified FCM model in Flow-3D software. To reduce compute time, the total number of cells is 160,000 and the width of grid is 2.2 mm, which has no significant difference from 800,000 cells in final simulation results. The mesh was shown in Figure 17.

The fluid material database of simulation software has provided most material thermal properties of Al 380, such as specific heat, thermal conductivity, latent heat of fluid and liquidus temperature. The material data can be directly used for simulation with only minor modifications. Here several important parameters such as melt temperature, barrel temperature and rotation speed of FCM process which have significant influence on microstructure of semisolid slurry were set in actual conditions. The rotational speed was 0~300 r/min and the corresponding simulation rotational speed was 0~18.84 rad/s due to speed control motors. The specific parameters and computation conditions used in the simulation are shown in Table 2.

## 6. Conclusions

A typical automotive part, a shock absorber part of A380 aluminum alloy, was produced by R-HPDC process with a new device of FCM machine for the preparation of semisolid slurry. From the analysis and discussion of the results presented in this study, the following conclusions can be drawn:

1. The microstructure at different locations of R-HPDC automotive shock absorber part are all composed of a large number of rosiness and nearly spherical primary α-Al particles. Due to the internal characteristics of semisolid slurry, the R-HPDC process can significantly improve porosity of castings not only in the quantity but also in the size. The R-HPDC process with FCM device decreases the total cross-sectional area of pore defects by about 27 times in one of the cross section.
2. Fine semisolid metal slurry with small, nearly spherical and uniformly primary particles distributed on the matrix can be obtained by adjusting the parameters of FCM device. Microstructure characteristics of semisolid A380 aluminum alloy slurry at different barrel temperatures and rotational speeds were investigated. Decreasing the barrel temperature can increase the cooling intensity of the device. As the barrel temperature decreases, the primary α-Al particles of the semisolid slurry increase in quantity and have been improved with the decrease of mean particle size and the increase of shape factor; with the increase of rotational speed, the mean size of the α-Al particles diminishes and the shape factor increases. When the barrel temperature is 540 °C and the rotational speed is 300 r/min, the average size of primary particles is about 60 μm and the shape factor is about 0.9, while the pouring temperature 610 °C and the mixing time of 30 s.
3. CFD results suggested that the axial flow and circular flow have encouraged the sufficient fluid convection in FCM process. The vigorous fluid convection has changed the temperature field and composition distribution of conventional solidification.
4. Rotational speed is directly related to the strength of fluid convection. Increasing the rotational speed can obviously improve uniform temperature distribution and reduce temperature difference around the grain within the same time, which destroy the generating environment of dendrites and benefit the generation of approximate spherical grain. Meanwhile the nuclei and the composition of the melt are constantly moving along with the flow and gradually uniformed distribution in semi-solid slurry, which will improve the unbalanced distribution of the composition.
5. The melt in FCM device is able to enter the semisolid temperature range faster and get a larger degree of supercooling with lower barrel temperature and pouring temperature, which can help increase the nucleation rate and decrease the grain size.

## Figures and Tables

**Table 1.** Mass chemical composition of aluminum alloy ingot.

Alloy	Chemical composition (wt%)						
A380	Cu	Si	Fe	Zn	Mg	Mn	Al
	3.18	8.47	1.05	1.58	0.09	0.27	Balanced

**Table 2.** Process and initial conditions of simulation.

Input	Value	Unit
Initial melt temperature	610	°C
Initial melt volume	500	mL
Initial barrel temperature	560	°C
Initial agitating shaft temperature	560	°C
Agitating shaft speed	6.28, 18.84	rad/s
Thermal conductivity of graphite barrel	129	w/m·K
Thermal conductivity of agitating shaft	23.4	w/m·K
Heat transfer to fluid	20	kw/m^2^·K

## References

[1] Zeren M. (2005). Effect of copper and silicon content on mechanical properties in Al-Cu-Si-Mg alloys. J. Mater. Process Technol.

[2] Lasa L., Rodrigues-Ibade J.M. (2003). Wear behavior of eutectic and hypereutectic Al-Si-Cu-Mg casting alloys tested against a composite brake pad. Mater. Sci. Eng. A.

[3] Mohamed A.M.A., Samuel A.M., Samuel F.H., Doty H.W. (2009). Influence of additives on the microstructure and tensile properties of near-eutectic Al-10.8%Si cast alloy. Mater. Des.

[4] Ejiofor J.U., Reddy R.G. (1997). Developments in the processing and properties of particulate Al-Si composites. JOM J. Miner. Met. Mater. Soc.

[5] Heinz A., Haszler A., Keidel C., Moldenhauer S., Benedictus R., Miller W.S. (2000). Recent development in aluminium alloys for aerospace applications. Mater. Sci. Eng. A.

[6] Miller W.S., Zhuang L., Bottema J., Wittebrood A.J., De Smet P., Haszler A. (2000). Recent development in aluminum alloys for the automotive industry. Mater. Sci. Eng. A.

[7] Verran G.O., Mendes R.P.K., Rossi M.A. (2006). Influence of injection parameters on defects formation in die casting Al12Si1,3Cu alloy: Experimental results and numeric simulation. J. Mater. Proces. Technol.

[8] Otarawanna S., Gourlay C.M., Laukli H.I., Dahle A.K. (2009). The thickness of defect bands in high-pressure die castings. Mater. Character.

[9] Avalle M., Belingardi G., Cavatorta M.P., Doglione R. (2002). Casting defects and fatigue strength of a die cast aluminium alloy: A comparison between standard specimens and production components. Int. J. Fatigue.

[10] Flemings M.C. (1991). Behavior of metal alloys in the semi-solid state. Metall. Trans. A.

[11] Ghomashchi R., Vikhrov A. (2000). Squeeze casting: An overview. J. Mater. Proc. Technol.

[12] Parshizfard E., Shabestar S.G. (2011). An investigation on the microstructural evolution and mechanical properties of A380 aluminum alloy during SIMA process. J. Alloys Compd.

[13] Kirkwood D.H., Suery M., Kapranos P., Atkinson H.V., Young K.P. (2009). Semi-Solid Processing of Alloys.

[14] Fan Z. (2002). Semi-solid metal processing. Int. Mater. Rev.

[15] Atkinson H.V. (2005). Modelling the semi-solid processing of metallic alloys. Prog. Mater. Sci.

[16] Wannasin J., Janudom S., Rattanochakul T., Canyook R., Burapa R., Chucheep T., Thanabumrungkul S. (2010). Research and development of gas induced semi-solid process for industrial applications. Trans. Nonferr. Met. Soc. China.

[17] Chung I.G., Molouri A., Kang C.G. (2012). A study on semisolid processing of A356 aluminum alloy through vacuum-assisted electromagnetic stirring. Inter. J. Adv. Manuf. Tech.

[18] Kang C.G., Kim S.K., Lee S.Y. (2006). Application of the continuous rheoconversion process to low temperature HPDC-Part II: Alloy development & validation. Solid State Phenom.

[19] Guo H.M., Yang X.J., Hu B. (2008). Low superheat pouring with a shear field in rheocasting of aluminum alloys. J. Wuhan Univ. Technol. Mater. Sci.

[20] Janudom S., Wannasin J., Basem J., Wisutmethangoon S. (2013). Characterization of flow behavior of semi-solid slurries containing low solid fractions in high-pressure die casting. Acta Mater.

[21] Chen Z.Z., Mao W.M., Wu Z.C. (2012). Preparation of semi-solid aluminum alloy slurry poured through a water-cooled serpentine channel. Inter. J. Min. Met. Mater.

[22] Wang N., Peng H., Wang K.K. Assessment of Porosity Level in Rheomolded Parts.

[23] Ji S., Fan Z., Bevis M.J. (2001). Semisolid processing of engineering alloys by a twin-screw rheomoulding process. Mater. Sci. Eng. A.

[24] Yang L.Q., Kang Y.L., Zhang F., Xu J. (2010). Microstructure and mechanical properties of rheo-diecasting AZ91D Mg alloy. Trans. Nonferr. Met. Soc. China.

[25] Seo P.K., Lee S.M., Kang C.G. (2009). A new process proposal for continuous fabrication of rheological material by rotational barrel with stirring screw and its microstructural evaluation. 209.

[26] Zhou B., Kang Y.L., Zhang J., Gao J.Z., Zhang F. Forced Convection Rheomoulding Process for Semisolid Slurry Preparation and Microstructure Evolution of 7075 Aluminum Alloy.

[27] Yang Y.T., Wang J.F., Zhang H.H., Shao G.J. (2008). The microstructure evolvement and simulation analysisi during semi-solid processing of aluminum alloy. Solid State Phenom.

[28] Ohno A. (1987). Solidification: The Separationtheory and Its Practical Applications.

[29] Jiang J.X., Wu Z.C., Chen L.L., Hao J. (2008). Numerical simulation and analysis of mould filling process in lost foam casting. China Foundry.

[30] Laukli H.I. (2004). High Pressure Die Casting of Aluminium and Magnesium Alloys Grain Structure and Segregation Characteristics. PhD Thesis.

[31] Verran G.O., Mendes R.P.K., Dalla Valentina L.V.O. (2008). DOE applied to optimization of aluminum alloy die castings. J. Mater. Process. Technol.

[32] Tseng C.H.E., Askeland D.R. (1992). Study of the EPC mold filling process using metal velocity and mass and energy balances.

[33] Faura F., Lopez J., Hernández J. (2001). On the optimum plunger acceleration law in the slow shot phase of pressure die casting machines. Int. J. Mach. Tool Manuf.

[34] Karban R. (2000). The Effects of Intensification Pressure, Gate Velocity & Intermediate Shot Velocity on the Internal Quality of Aluminum Die Castings. PhD Thesis.

[35] Brungs D. (1997). Light weight design with light metal castings. Mater. Des.

[36] Niu X.P., Hu B.H., Pinwill I.P., Li H. (2000). Vacuum assisted high pressure die casting of aluminum alloys. J. Mater. Process Technol.

[37] Fan Z., Fang X., Ji S. (2005). Microstructure and mechanical properties of rheo-diecast (RDC) aluminum alloys. Mater. Sci. Eng. A.

[38] Zhao J.W., Wu S.S. (2010). Microstructure and mechanical properties of rheo-diecasted A390 alloy. Trans. Nonferrous Met. Soc. China.

[39] Mahallawi E., Abdelkader H., Shehata L., Amer A., Mayer J., Schwedt A. (2013). Influence of nanodispersions on properties and microstructure features of cast and T6 heat-treated Al Si hypoeutectic alloys. Solid State Phenom.

[40] Möller H., Govender G. (2013). The heat treatment of rheo-high pressure die cast Al-Cu-Mg-Ag alloy 2139. Solid State Phenom.

[41] Bouzakis K.D., Maliaris G., Tsouknidas A. (2012). FEM supported semisolid high pressure die casting process optimization based on rheological properties by isothermal compression tests at thixo temperatures extracted. Comput. Mater. Sci.

[42] Modigell M., Koke J. (2001). Rheological modeling on semisolid metal alloys and simulation of thixocasting processes. J. Mater. Proc. Technol.

[43] Song W.X. (1989). Metallography.

[44] Zhang B.J., Cui J.Z., Lu G.M. (2003). Effect of low-frequency magnetic field on macrosegregation of continuous casting aluminum alloys. Mater. Lett.

[45] Samanta D., Zabaras N. (2005). A coupled thermomechanical, thermal transport and segregation analysis of the solidification of aluminum alloys on molds of uneven topographies. Mater. Sci. Eng. A.

